# Designed Fibril-Forming Mini-Collagens Engineered to Exhibit up to Two Orders of Magnitude Differences in Rates of Matrix Metalloproteinase I Susceptibility

**DOI:** 10.1021/acs.biomac.5c00026

**Published:** 2025-06-23

**Authors:** Jui Shivaji Chaugule, Yujia Xu

**Affiliations:** 1 The Graduate Center, Program of Biochemistry, The City University of New York, 365 Fifth Ave., New York, New York 10016, United States; 2 Department of Chemistry, Hunter College of the City University of New York, 695 Park Ave., New York, New York 10065, United States

## Abstract

The susceptibility to matrix metalloproteinases (MMPs) directly affects the functions and applications of collagen biomaterials. In this work, we demonstrated that this property can be manipulated in collagen-mimetic biomaterials created using designed peptides. We developed three fibril-forming mini-recombinant collagens (MRCs) using bacterial expression and designed genes that model a 108-residue section of human type III collagen surrounding the MMP-1 recognition site. Notably, the MRCs can form a native-like fibrillar structure representing the natural substrate of MMP-1. By altering the number of digestion sites or mutating the residues at the canonical scissile bond of MMP-1, the sensitivity to proteolysis of the MRCs varied by two orders of magnitude despite having homologous amino acid sequences and a similar fibrillar structure, and regardless of whether the peptides were in the triple helix conformation or as fibrils. These MRCs can be a versatile collagen alternative for regenerative medicine offering a regulated turnover rate catering to specific applications.

## Graphical abstract

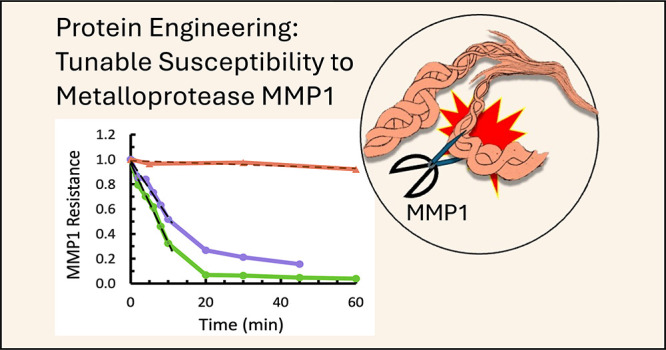

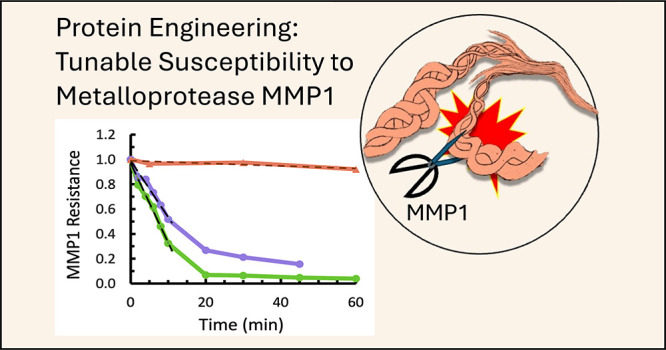

## Introduction

One of the major advantages of collagen-mimetic materials produced using designed peptides is the feasibility to optimize the biological functions of the materials by controlling their amino acid composition. Collagen-based and collagen-mimetic biomaterials have found a wide range of medical applications such as wound healing, bone grafting, and cosmetic surgeries.^1–7^ The collagenous component of the materials is designed to function as a temporary molecular scaffold at the site of tissue damage to facilitate the migration and the adhesion of host cells.^7–10^ Ideally, these collagen-based molecular scaffolds should resemble that of the host extracellular matrix (ECM) in both chemical composition and mechanical stiffness. Once the cells start to spread and to differentiate at the site, the scaffold should be gradually degraded and eventually be eliminated to make room for the nascent ECM created by the host cells. The homeostasis of the ECM during tissue remodeling critically depends on the delicate balance between new collagen production and the degradation of defective and/or redundant collagen scaffold.^3,4,11–13^ The rate of collagen-mediated tissue repair varies from minutes during hemostasis to months for wound maturation and bone regeneration.^3,7^ Medical devices used for these applications should be stable enough to aid the tissue remodeling without the needs of frequent reapplications, and yet be responsive to the necessary collagenolytic reactions to avoid overaccumulation of fibrous materials, which are often associated with fibrosis and scar tissues.^12,13^ The timed turnover of collagen materials to match the progression of the natural pace of tissue repair is thus an important element to engineer in order to achieve the optimal performance of the collagen-based medical devices during applications.

The major component of the molecular scaffold of the ECM is fibrillar collagen, mainly collagen types I, II, and III. Collagen monomers first form the rod-shaped triple helix consisting of three parallel polypeptide chains twisted together about a common axis. Each polypeptide chain has more than 1000 amino acid residues in a strict Gly-X-Y repeating amino acid sequence, where the X and Y can be any amino acid residues. The three polypeptide chains in collagen type II and type III are identical, while the type I collagen is a heterotrimer in which one of the three polypeptide chains has a different amino acid sequence from the other two. The triple helices further self-assemble laterally with a mutual staggering of 64 nm at the ends to form fibrils with the characteristic axially repeating structure known as the *D*-period.^14–18^ The specific molecular interactions of the side chains of the X- and Y-residues determine the structural specificity of the *D*-periodic molecular packing. The *D*-period is one of the major structural elements responsible for the unique molecular and mechanical properties of collagen fibrils. The side chains of critical residues on the surface of the fibrils interact with cell receptors and/or enzymes, while the structure of the fibril itself provides the critical stiffness and the mechanical cues to modulate these interactions. The turnover of fibrillar collagen in tissues involves several matrix metalloproteinases (MMPs) with the matrix metalloproteinase-1 (MMP-1, also known as collagenase 1) being the major catabolic enzyme in skins and bones.^19–21^ All three fibrillar collagens share the same MMP-1 digestion site located about 225 nm from the N-terminus of the triple helix.^22–26^ MMP-1 digestion cleaves the triple helix into two distinctive 3/4- and 1/4-length fragments. While the triple helix is the preferred substrate of MMP-1 *in vitro*, the fibrillar form of collagen is the physiological substrate in tissues.^27–31^ After latching onto the fibrils, MMP-1 moves unidirectionally along the axis of the fibrils and make cuts at the specific sites.^32,33^ How accessible are the cleavage sites in the fibrils and what are the conformational changes of collagen fibrils after each cleavage action are still under investigation.^34–36^

The highly regulated interactions of collagen with MMPs affects the tissue development and remodeling in more ways than one; some of the molecular events are still not fully understood.^37^ During the wound healing, the rate of collagen degradation changes during the process, starting out at a relatively slow rate during the early stages of the process and increasing as wound maturation occurs, which is accompanied by the increased expression of MMP-1 by cells in the wound.^3,11^ During the activation of hemostatic pathways at the onset of a cutaneous wound, the interactions of collagen with platelets trigger the coagulation pathways and at the same time promote the release of glycoproteins and growth factors that are chemotactic for fibroblasts and other connective tissue cells. A stable fibrillar structure in this stage promotes the proliferation of the cells. The anchored fibroblasts will, then, undergo a period of rapid synthesis of type I and type III collagen and increase the tensile strength of the wound. While normal skin consists of 80–90% type I collagen and 20–10% type III collagen, the ratio of type III collagen increases during the early stages of wound healing (first 24 h to 2 days), highlighting the potential critical roles of type III collagen in facilitating the seeding and attachment of fibroblasts.^11^ As the stage of neo-matrix formation progresses, the catabolism of collagen starts to pick up. New MMP-1 is secreted by the tissues to balance the large-scale fibroplasia. The activity of MMP-1 in this process is tightly regulated by both the MMP-1 activation protease such as plasmin and the MMP-1 inhibitor such as α2-macroglobulin. Fibroproliferation without the adequate catabolism in this stage is the major cause of imperfect ECM as seen in the development of scar tissues or keloid body.^4,11,38^ Controlling rapid deposit of the collagenous materials that could be better balanced by the degradation and/or activation of the activity of MMP-1 has been the focus of approaches for more functional ECM.^3,39^ Medical devices that can facilitate this process are expected to significantly improve wound quality.

The collagen-based therapies utilize collagens sourced from animals or human allograft from cadavers. The concerns of cross-species transmission have limited the use of animal collagens, while the products from cadavers suffer from limited availability and reproducibility.^7^ Since both collagen and MMPs are highly conserved in mammals, the tissue-derived collagens, regardless of the source, all have similar inherited susceptibility to MMP-1 and other collagenases that are difficult to alter or eliminate. The protein-engineered collagen-mimetic peptides suggest a potential means to manipulate the protease susceptibility by selecting specific amino acid sequences.^40^ A major challenge remains, however, because these designed peptides often do not form native-like fibrils without extensive chemical modifications, which limited their functionality in medical applications.^41,42^ In recent years, we have developed fibril-forming mini-recombinant collagens (MRCs) that, upon forming stable collagen triple helices, can further self-assemble into fibrils having *D*-period like axially repeating structures based on similar molecular interactions observed in native fibrillar collagens.^43–46^ The native-like fibrillar structure adds a new dimension to the applications since the fibrils can provide a more native-like molecular scaffold. In this work, we created three new MRCs that have varied MMP-1 susceptibility. The peptides consist of sections of 108 amino acid sequences modeling the region in human type III collagen surrounding the MMP-1 digestion site. The prominent involvement of type III collagen during the early onset of wounds makes it a particular interesting subject to study to understand the homeostasis of the ECM during the process. The homotrimeric nature of type III collagen also makes it relatively easier to model using the MRCs.

## Materials and Methods

### Peptide Biosynthesis

The genes of the three peptides were synthesized using GenScript services, sequence-optimized for bacterial expression, and cloned into modified pET32a­(+) plasmids, as described in the previous work.^43^ The three peptides were expressed in strain BL21­(DE3) as a fusion protein with a His-tagged thioredoxin (Trx) at the N-terminus. The expression and purification followed the procedures described previously.^43^ Briefly, the cells were first grown in Amp+ LB medium at 37 °C and induced with IPTG when OD_600_ reached 0.6. The final concentration of IPTG is 0.1 mM. After induction, the medium was transferred to a shaker incubator set at 16 °C and grown overnight (∼16 h) at 16 °C/225 rpm.

### Purification Using His-Tag Affinity Chromatography

The cells were harvested first by centrifugation at 4000 rpm for 20 min at 4 °C, and the pellet was resuspended in prechilled Tris buffer (50 mM Tris, 300 mM NaCl, pH 7.4). The cells were ruptured by sonication (Vibracell, six 3 s pulses, machine output 30, duty cycle 50%) and centrifuged again at 7000 rpm for 28 min at 4 °C. The supernatant was retained for His-tag affinity chromatography using HisPur cobalt resin (Thermo Fisher, 89964) and eluted with 500 mM of imidazole following the procedures in the manufacturer’s manual. The eluents were collected and dialyzed to remove the imidazole using 3 MWCO dialysis cassettes with a molecular cutoff of 3 kDa (Thermo Fisher, 66110). The dialysis was carried out at 25 °C against the Tris buffer (pH 7.4) in a volume ratio of 5:300 with constant stirring. The buffer was changed twice after every 4 h and then left to continue overnight (∼16 h). The His-Trix tag was removed by thrombin cleavage using human thrombin enzyme (Thermo Fisher, T6884). The enzyme (0.324 μg) was first dissolved in 100 μL of Millipore water and was added during the dialysis in a ratio of thrombin to peptide of 1:500.

### Purification by HPLC

The dialysate was further purified using HPLC to remove the His-Trix tag as described in ref 43 using a reversed-phase C8 Semiprep column (Vydac, 208TP1010). The peptide was eluted at ∼43% acetonitrile, and the fractions corresponding to the peptides were collected and lyophilized. The purified peptides were stored as lyophilized powder until use.

### Sample Preparation

The peptide samples were made by first dissolving in 5 mM acetic acid (HOAc, pH 3.9) at a concentration of about 4 mg/mL by weight and equilibrated in the refrigerator for at least 7 days. The concentrations were further calibrated by OD_280_ using a NanoDrop, or a UV–vis spectrometer before use; the extinction coefficient was calculated to be 0.32 mL/mg using the online tool ProtParam. Each peptide has 292 amino acid residues and a molar weight of 27.098 kDa. The same parameters are applicable to all three peptides because of their nearly identical amino acid sequences.

### The Circular Dichroism (CD) Spectra of the Peptide

The CD spectra were taken using a Chirascan V100 spectrometer by Applied Photophysics. The scans were taken at 4 °C using a 1 mm quartz cuvette. The spectra of peptide samples (∼0.2 mg/mL in concentration) were corrected with the blank scan of corresponding buffer using the same cuvette. The raw CD data in millidegree was normalized and converted to the mean residue molar ellipticity (MRE) using the equation:

$$[\theta ]=\frac{\deg\times m}{c\times l\times n_{r}}$$

where deg is the raw data in millidegree, *m* is the molar weight of the peptide (single chain), *c* is the concentration of the peptide in mg/mL, *l* is the optical path in cm, and *n*_r_ is the number of residues per single peptide chain.

### Thermal Stability Characterization

For the temperature melt experiment, the CD spectra over the range of temperature between 5 and 60 °C were monitored by setting the heating rate at 1 °C/min with a temperature step of 1 °C and an equilibration time of 20 s at each temperature. The fraction of folded (FF) at each temperature (*T*) was calculated using the equation:

$$\mathrm{FF}(T)=\frac{\theta (T)-\theta_{\mathrm{mono}}(T)}{\theta_{\mathrm{tri}}(T)-\theta_{\mathrm{mono}}(T)}$$

where θ­(*T*) is the CD signal at 225 nm at *T* in MRE and θ_mono_(*T*) and θ_tri_(*T*) are the extrapolated values of the CD signal of the unfolded monomer and the folded triple helix in MRE, respectively. The baseline of monomer was determined from a linear fit of CD signals at the temperatures between 55 and 60 °C and that of the triple helix from the CD signal between 5 and 15 °C. The *apparent* melting temperature (*T*_m_) was determined as the midpoint of the sigmoidal curve using the Boltzmann sigmoidal equation of SciDAVis, which is equivalent to the temperature of FF equals 0.5 assuming that the curve is perfectly symmetric. For the temperature melting experiment, the concentration of the peptides is ∼0.4 mg/mL in 5 mM HOAc using a 1 mm cuvette.

### *In Vitro* Fibrillogenesis

In vitro fibrillization was initiated by mixing samples of peptides in 5 mM HOAc with an equal volume of 2× TES buffer (60 mM TES, 50 mM Na_2_HPO_4_, and 135 mM NaCl, pH 7.4) or, for the experiments of MMP-1 digestion, 2× Tris buffer (300 mM Tris, 400 mM NaCl, and 20 mM CaCl_2_, pH 7.5) and incubated at the specified temperature (between 25 and 34 °C) for 4 h to 2 weeks.

### Transmission Electron Microscopy (TEM)

The fibril structure was analyzed by TEM using the JEOL JEM-2100 spectrometer at the Imagine Facility of the City College of New York, the City University of New York (CCNY), or the Advanced Science and Research Center, the City University of New York (ASRC). The 300 mesh copper grids were purchased from Ted Pella, Inc. Five microliters of sample was placed on the grid for 60 s followed by adding 2% fresh phosphotungstic acid for 30 s. The staining was repeated twice (let it dry or blotted dry). No washing was done in between the staining. The grid was air-dried for 30 min before imaging. For some of the images, the grid was plasma cleaned before using with the Fishione NanoClean M1070 to increase the hydrophilic property of the grid.

### MMP-1 Digestion of Colt3 Peptides

The human MMP-1 was purchased from Sigma (cat no. SRP3117). Each package is 10 μg based on the product specification. A stock solution of 23.8 μM was prepared by dissolving all 10 μg of MMP-1 in 595 μL of 2× Tris buffer. The digestion assay was performed by mixing 5 μL of MMP-1 stock solution with 5 μL of collagen stock solution in 5 mM HOAc such that the final concentration of MMP-1 in the reaction mixture was 0.2 μM, and that of collagen varied between 2.5 and 14.7 μM (0.2 to 1.2 mg/mL). The pH after mixing was 7.0 confirmed by a mixing test using the same buffers in the same mixing ratio. Ten-microliter reaction mixtures were made for each time point. The mixtures were quickly vortexed and incubated in a 32 °C water bath immediately. At each desired time point, a reaction mixture was taken out, and the reaction was stopped by adding 1 μL of EDTA to a final concentration of 25 mM, and 2 μL of 5× SDS (with 0.2 M DTT) to a final concentration of ∼1× SDS and heated in a water bath at 95 °C for 30 min. The samples were then analyzed by SDS-PAGE using 4–20% gradient gel (GenScript M00654) or 12% gel made in-house. Pictures of the gel images were taken using a digital camera or mobile devices. The pictures (in JPG or TIFF format) were then converted into densitometry plots using the gel analysis function of ImageJ.^47^

### MMP-1 Digestion of Fibrillar Colt3 Peptides

Colt3 peptides in 5 mM acetic acid was allowed to form fibrils by mixing with 1 M Tris (pH 7) in a 1:1 ratio. This mixture was aliquoted in 5 μL volumes on ice for various time points and then incubated at 32 °C for 24 h to allow the formation of fibrils. The digestion assay was performed by adding 5 μL of 0.4 μM MMP-1 to each 5 μL aliquot of fibril samples and incubated in a 32 °C water bath. The final concentration of MMP-1 in the reaction mixture was 0.2 μM. After each time point, the reactions were stopped as described above. The enzymatic assay was carried out for Colt3_1 mini-fibrils at final concentrations of 1.2, 6.1, and 12.3 μM, and Colt3_2 fibrils at final concentrations of 1.2, 3.7, and 6.1 μM. For the Colt3_0 peptide, the reaction was carried out only for one concentration, 14.7 μM. The SDS-PAGE analysis and the densitometry data were read using the gel function of ImageJ, as described above.

## Results and Discussion

### The Colt3 Peptides

The mini-recombinant collagens (MRCs) for this study are designed using the same strategy of our previous work.^43,44,46^ Collectively, the three MRCs are designated as the Colt3 peptides: Colt3_0, Colt3_1, and Colt3_2. As depicted in Figure 1A, the peptides consist of two identical sequence units (SU1 and SU2) interspersed with (Gly-Pro-Pro)_4_ repeating sequences (GPP_4_). Each sequence unit consists of 108 amino acid residues; the entire triple helix domain is 252 residues long in noninterrupted Gly-Xaa-Yaa repeating sequence. The 27-residue foldon domain at the C-terminus is included to serve as the nucleation domain for trimerization. A Cys-knot sequence consisting of residues Gly-Pro-Cys-Cys is included at the N- and C- termini of the triple helical domain for potential interchain covalent cross-linking. All three peptides are identical in size but differ in amino acid sequences.

**Figure 1:**
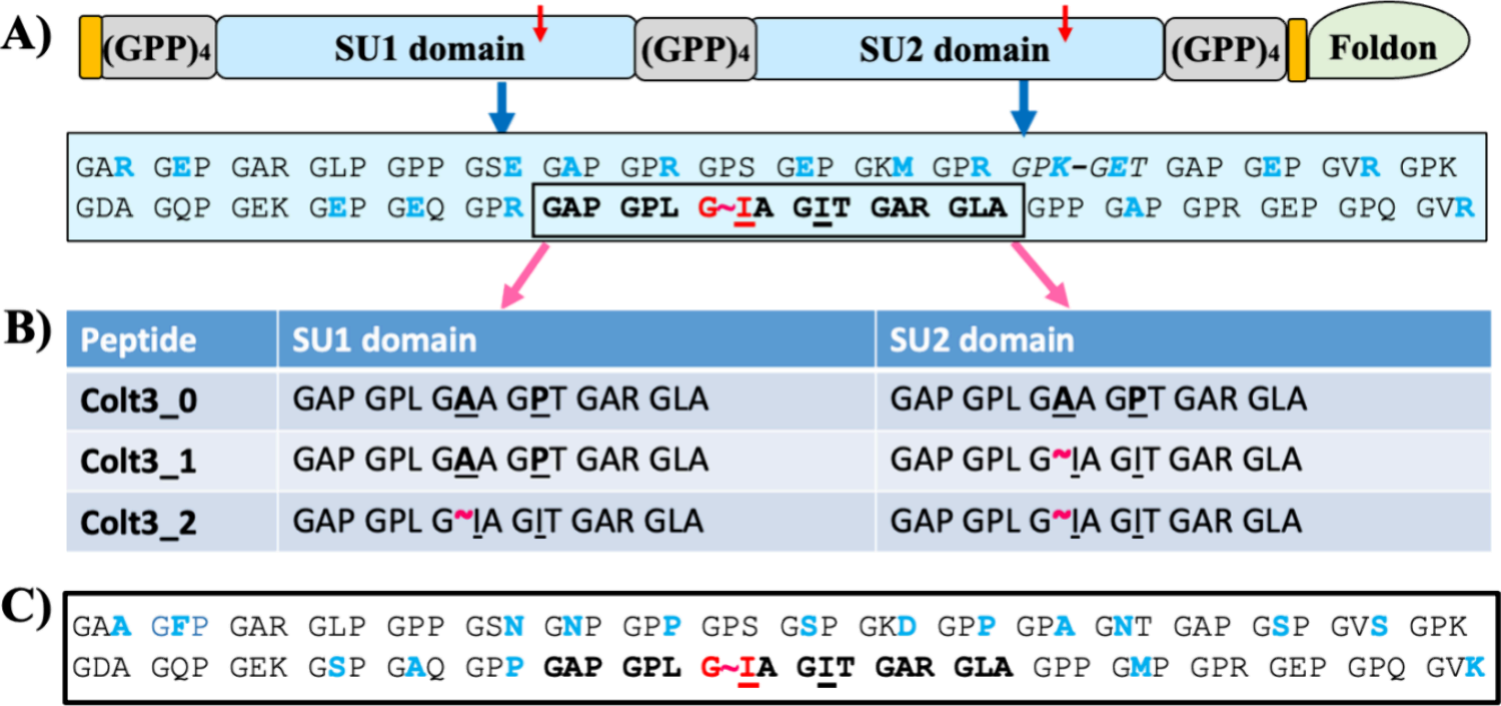
Amino acid sequence architecture of Colt3 peptides. (A) Schematic diagram highlighting the major design features of peptide Colt3_2 with the amino acid sequence of the identical SU1 and SU2 domains shown in the text box. The amino acid residues of the MMP-1 recognition site are boxed; the scissile bond between a Gly and an Ile is highlighted by a red tilde, and their corresponding locations in Colt3_2 are marked by red arrows. The residues that are subject to substitutions in peptides Colt3_0 and Colt3_1 are underscored. An inserted KGE (Lys-Gly-Glu) tripeptide is italicized and connected with a dash (see text). The two orange blocks mark the locations of the Cys knot. (B) Amino acid sequences corresponding to the MMP-1 recognition site in SU1 and SU2 domains of the three Colt3 peptides; the residues that are varied in the three peptides are underlined and highlighted in bold, and the expected MMP-1 digestion sites marked by the red tilde. (C) Amino acid sequence of residues 768–875 of human type III collagen with the modified residues shown in blue (see the text).

The amino acid sequences of SU1 and SU2 were selected to model the section of 108 amino acid residues (residues 768–875) around the sole MMP-1 digestion site of human type III collagen. The 18 residues between Gly^843^ and Ala^857^ are identified as the MMP-1 recognition site with the scissile bond located between Gly^846^ and Ile^847^ (Figure 1A).^48^ This selected region includes several residues having relatively low triple helix propensity according to Persikov et al.^49,50^ To ensure that the triple helix would have good thermal stability, some of the residues outside of the MMP-1 recognition site were replaced by those having higher propensity for the triple helix conformation. Several Ser, Asn, and Phe residues in the X-position were substituted by Glu or Ala (Figure 1A,C); the Lys and Ser in the Y-position in the triplet Gly-Val-Y were substituted to Met or Arg. Among the stabilizing factors, the sequence Lys-Gly-Glu (KGE) has the most profound impact through a set of interchain salt bridges.^51,52^ For this reason, a KGE sequence was included by replacing the Ala and Asn in the sequence of Gly-Pro-Ala-Gly-Asn-Thr to Lys and Glu, respectively. Finally, to keep the pI of the peptide close to that of the native type III collagen, several Pro residues in the Y-position were replaced by Arg. While the exact roles of charged residues are not fully elucidated, the fibrillogenesis depends on both pH and ionic strength of the buffer. The charged residues are thus expected to be involved in both the molecular recognition and the stabilization during the process. Arg in the Y-position has slightly lower triple-helix propensity than Pro. The Pro to Arg substitutions in the selected positions are not expected to have a significant effect on the *overall stability* of the triple helix. The ring structure of a Pro residue, however, affords a unique rigidity of the peptide backbone. These substitutions outside of the MMP-1 recognition site are also expected to not affect the functions of MMP-1.^48^

Among the three Colt3 peptides, peptide Colt3_2 retained the MMP-1 recognition site in both SU1 and SU2 and thus has two MMP-1 susceptible Gly-Ile bonds (Figure 1A). Peptide Colt3_1 is designed to have only one MMP-1 digestion site located in the SU2 by replacing the Ile^847^ in the SU1 with an Ala. The Ile^850^ of the SU1 was also replaced with a Pro to prevent it from acting as an alternative MMP-1 cutting site in the absence of Ile^847^ (Figure 1B).^48^ In Colt3_0, the Ile^847^ and Ile ^850^ in both SU1 and SU2 are substituted to create a peptide having no *known* MMP-1 cleavage site. It is therefore expected that Colt3_2 has the highest susceptibility to MMP-1 among the three peptides, while Colt3_0 is resistant to proteolysis by MMP-1.

The three peptides were expressed in and purified to a high degree of purity (Figure 2A). The monomer of the peptides has a molecular weight of 27.6 kDa, which migrates to just below the 35 kDa molecular marker. Moving above the expected location on SDS-PAGE is a known phenomenon of collagen peptides, presumably due to its relatively stiff backbone consisting of high content of Pro residues.^44^ Similarly, the trimer and dimer forms also migrated to locations slightly higher than what were expected from their actual molecular weight: between 65 and 95 kDa markers for the dimer (when visible) and above 130 kDa for the trimer. The oligomers often appear as multiple bands due to variations in the compactness of the denatured conformation having different degrees of cross-linking.^44^ The complete reduction of all disulfide bonds is difficult even in high concentrations of DTT (0.5–1 M). Some of the oligomers are cross-linked by the disulfide bonds at the C-terminus, while others at the N-terminus or both. These peptides will experience different levels of unfolding in the presence of SDS and heating and contribute to different mobilities on the gel. Similar results are seen for all three peptides because of their nearly identical amino acid sequences.

**Figure 2:**
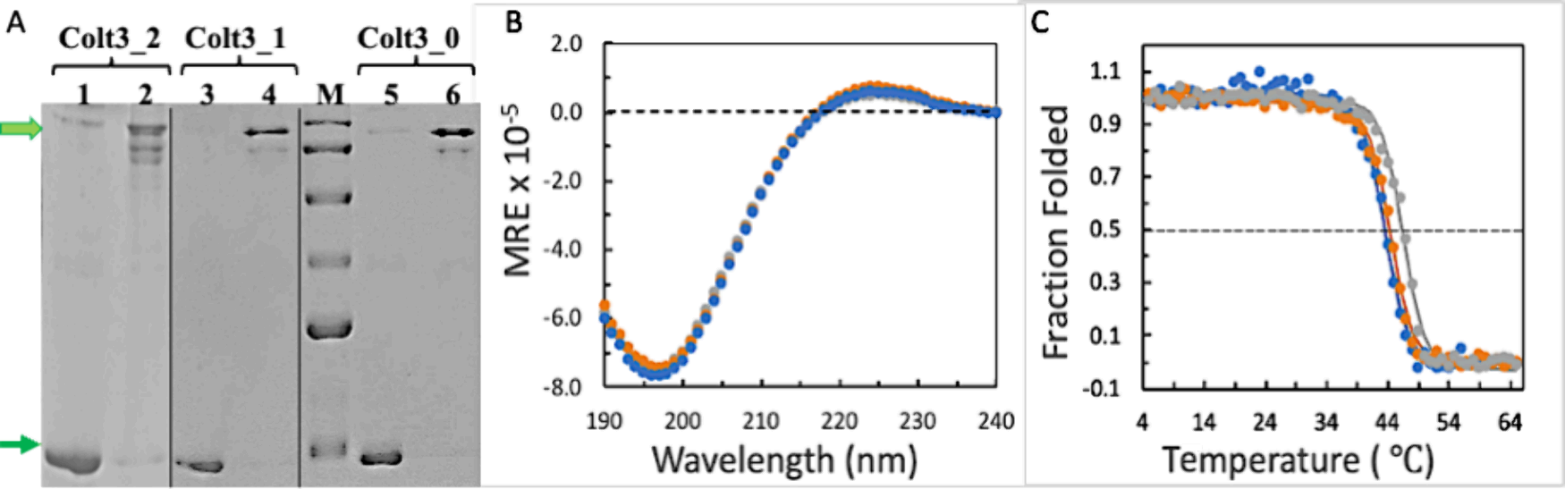
The folding and the thermal stability of Colt3 peptides. (A) Purity of the Colt3 peptides was monitored by SDS-PAGE: lanes 1, 3, and 5 are samples reduced with 0.5 M DTT; the trimer and monomer are marked by thick and thin green arrows, respectively; lane M is the molecular marker (from top to bottom: 175, 130, 95, 65, 50, and 35 kDa). The picture includes the results of three separate gels separated by the thin vertical lines. The loading concentrations are 1 μg per well for Colt3_0 and Colt3_1, and 2 μg per well for Colt3_2. (B) CD spectra of Colt3_0 (gray), Colt3_1 (orange), and Colt3_2 (blue) in 5 mM HOAc at 4 °C. (C) Temperature melting curve of the three peptides in 5 mM HOAc in the same color scheme as in (B). The solid lines are drawn using the Boltzmann sigmoidal equation of SciDAVis.

The fact that the peptides present predominantly as a trimer under nonreducing conditions is an indication that the peptides form a well-aligned triple helix before subjected to SDS, since the set of disulfide bonds of Cys knots can only form in the structural context of a triple helix.^46,53,54^ Upon reduction, all bands in the high molecular region disappeared and were replaced by a single strong band of monomer. The triple helix conformation of the three peptides is further confirmed by the CD spectra. The positive peak at 225 nm together with the deep negative peak at 197 nm is characteristic of a triple-helix conformation. The spectra of the three peptides are nearly identical due to the high degree of sequence homology. Similarly, the temperature melt experiments revealed similar apparent melting temperatures of the three peptides. The estimated apparent *T*_m_ are 43.4, 44.3, and 46.4 °C for peptides Colt3_2, Colt3_1, and Colt3_0 (Figure 2C), respectively, with Colt3_0 being the most stable one.

How to increase the thermal stability of the bioengineered, fibrillar-form collagen-mimetic peptides remain a challenging aspect. The triple-helix conformation is intrinsically unstable despite its *rod-like* reputation. The thermal stability of native collagen triple helices are often a few degrees lower than the physiological temperature of the organism.^55^ Short, synthetic peptides can reach higher thermal stability because of their exceedingly high Gly-Pro-Hyp (Hyp = hydroxyproline) content and short chain length.^56^ The same two factors, on the other hand, also limited the functionality and the broader perspectives of the synthetic peptides as biomaterials. Like other MRCs generated in our lab, by combining the stabilizing Cys knot(s) and choosing residues with high triple-helix propensity, the Colt3 peptides have reached *T*_m_ values above 42 °C, which is suitable for routine laboratory processing. The *T*_m_ of tissue-derived type III collagen, in comparison, is between 36 and 37 °C. The triple helix propensity of amino acid residues obtained from the host–guest peptides, albeit not applicable in the quantitative sense, has proven to be a robust guide on predicting the stabilizing impact of individual residues.^49,50^ The thermal stability between the three peptides is small but observable: the apparent *T*_m_ values of Colt3_1 and Colt3_0 are higher than those of Colt3_2 by ∼1 and 3 °C, respectively. The amino acid sequence of Colt3_1 differs from that of Colt3_2 by the substitutions of Ile to Pro and Ile to Ala, in SU1, while Colt3_0 has the same two substitutions in both SU1 and SU2. Ile in the X-position is one of the most destabilizing residues for the triple-helix conformation when tested in the host–guest peptides.^49^ The increased *T*_m_ is consistent with the Ile substitutions, and the degree of increase appears to correlate with the number of substitutions.

### 2 The Fibrillogenesis of the Colt3 Peptides

All three peptides form similar fibrillar structures upon incubation at 34 °C in buffers at neutral pH and containing salt (NaCl) (Figure 3). The fibrillar structure shows a similar morphology as the native collagen fibrils having long and flexible appearances with uniform diameter and tapered ends. The positively stained regularly spaced fine bands are consistent with the design to have the triple helices self-associate laterally with the mutual stagger of one sequence unit.^43–46^ Because of the same amino acid sequences in SU1 and SU2, the unit staggering will bring the clusters of positively and negatively charged residues in associating triple helices into register. The clusters of positively charged residues interact with the heavy metal ions of phosphotungstic acid staining solution to appear as dark bands. The persistent presence of the same banding pattern of fibrils from all three peptides and fibrils formed in different solvent conditions and at different temperatures point to the sequence-unit staggering as the unified underlying mechanism of fibrillogenesis. The similar positive staining pattern, characterized by regularly placed finer bands associated with the in-register alignment of charged residue clusters, also serves as a hallmark feature of the *D*-period fibrillar structure of native collagen fibrils.^57,58^ The fibrils of Colt3 peptides are rather thin, however, with an average diameter about 25 nm. As a result, the 35 nm *d*-period of the gap overlap was only observed in occasions when fibril diameters reached ∼50 nm (Figure 3A).^43,46^

**Figure 3:**
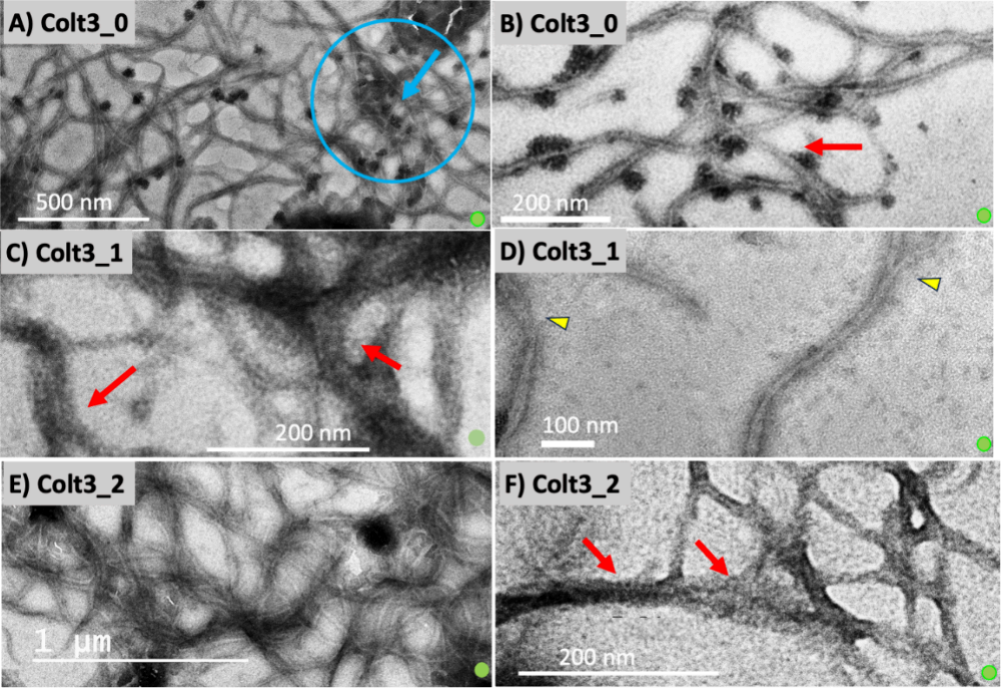
The fibrillar structure of Colt3 peptides. The fibrils of Colt3_0 (A, B), Colt3_1 (C, D), and Colt3_2 (E, F) are formed by incubating the peptides in fibrillar buffer(s) at neutral pH and at 34 °C except B, which was incubated at 26 °C. Red arrows mark where the positively stained banding patterns are visible, and the blue circle and arrow indicating a region with negatively stained effect; the yellow triangle marked the merging point of fibrils. The small green spheres are included as an indicator of the correct aspect ratio.

In addition to the similar fibril structure from the lateral assembly of regularly staggered triple helices, the fibrillar structure of the MRCs is also stabilized by the similar molecular interactions as those observed in native collagen. The fibrillogenesis of the peptides showed similar temperature and salt dependence as that observed during the in vitro fibrillogenesis of tissue-derived type III and type I collagens.^59^ The fibrillogenesis is favorable at higher temperatures; at 26 °C, the fibrillar structures do form, but they are thinner and shorter compared to those obtained at 34 °C (Figure 3B) after up to 2 weeks of incubation. The same positively stained banding pattern, however, indicates that it is the same assembly process albeit proceeding at a slower pace. The fibrillogenesis of native collagen requires 150–200 mM of salt.^59^ Similarly, no signs of fibrillar assemblies of the MRCs were observed in pH 7 buffer without added salt. The fibril diameters are likely limited by the lower concentration used for fibrillogenesis study of the MRCs. The yield of bacterial expression without using a fermenter was low, and the nature of enzymatic degradation experiments has put a tremendous pressure on the availability of peptides for more extensive structural studies. Studies of other MRCs produced in our lab at higher concentrations have routinely produced fibrils with the diameter in the range of 50–75 nm, which overlaps with the range of diameters of fibrillar collagens in skin and other softer tissues.

### 3 The Different Susceptibility of Colt3 Peptides to MMP-1

The MMP-1 digestion of the Colt3 peptides will create well-defined fragments determined by the location(s) of the scissile bond(s). Having one MMP-1 recognition site, the Colt3_1 peptide should be cleaved into two fragments: an N-terminal fragment having a molecular weight about 20 kDa (N20) and a C-terminal fragment about 7 kDa (C7) (Figure 4A). The digestion of the Colt3_2 peptide, which has two MMP-1 recognition sites, can generate two to five fragments (Figure 4A). The cleavage at the C-terminal MMP-1 site alone will create the same N20 and C7 fragments similar as the digestion of Colt3_1, while those at the N-terminal MMP-1 site would create an 8 kDa N-terminal fragment (N8) and a 19 kDa C-terminal fragment (C19). If the enzyme acted on both sites, either simultaneously or sequentially, then it would generate a new fragment of about 12 kDa (M12) plus the N8 and C7 fragments. In contrast, no fragments were expected from the MMP-1 digestion of Colt3_0 since the peptide is expected to be resistant to degradation for having no MMP-1 recognition site.

**Figure 4:**
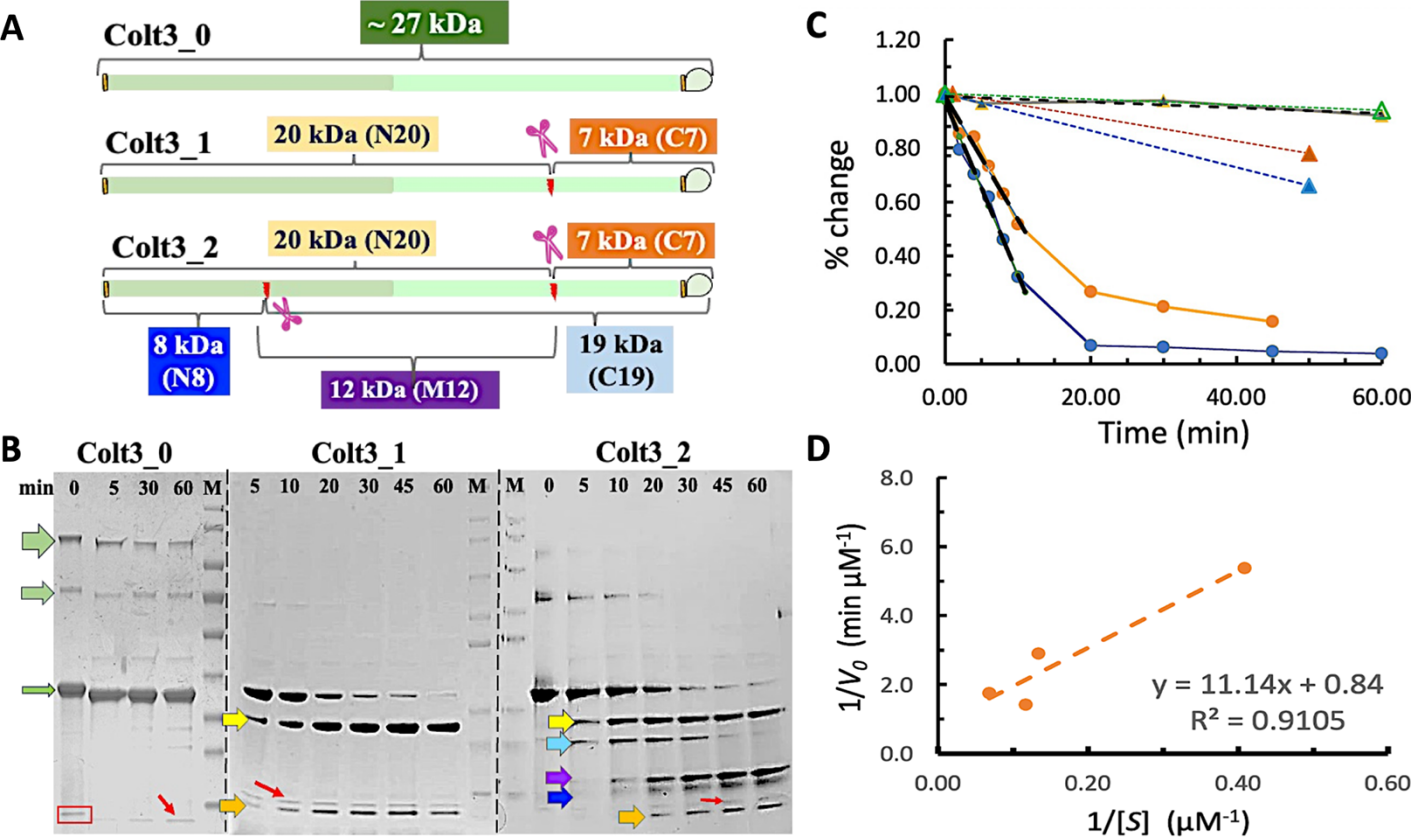
The MMP-1 digestion of Colt3 triple helices. (A) Schematic drawing showing the digestion sites of the three peptides and the degradation fragments. (B) Proteolysis of the three peptides monitored by SDS-PAGE using 4–20% gradient gel (Colt3_1 and Colt3_2) or 12% gel (Colt3_0). The numbers on the top of the lanes are the time of incubation in minutes; lanes labeled M are molecular markers in kDa. The three green arrows in increasing width represent, respectively, the monomer, dimer, and trimer forms of noncleaved peptides. The digestion fragments are marked using arrows in the same color scheme as in (A). The vertical dashed line marks the boundary of three separate gels. The red box and arrows indicate the possible impurity that does not change during the incubation with enzyme. The concentration of MMP-1 is 200 nM, and lane M is the molecular makers (from top to bottom: 270, 175, 130, 95, 65, 50 35, 30, and 15 kDa). (C) Rates of degradation of Colt3_0 (gray), Colt3_1 (orange), and Colt3_2 (blue). The solid lines connecting the data points are included as a guide to the eye. The linear fit of the data used to estimate the initial rate is shown as dashed lines. The concentrations of Colt3_1 and Colt3_2 are 7.40 μM, while that of Colt3_0 is 14.7 μM (see text). For easier comparison, we also included the digestion data of fibrillar form of Colt3_2 (solid blue triangle), Colt3_1 (solid orange triangle), and Colt3_0 (open green triangle). The dotted lines connecting the data points are included as guide to the eye. For more details, see text and Figure 5. (D) Lineweaver–Burk plot of the MMP-1 digestion of triple helical Colt3_1 at 32 °C.

The MMP-1 digestion experiments were first carried out for the three peptides in the triple-helix conformation by preincubating the peptide in pH 3.9 buffer and at low temperature to prevent fibril formation before the addition of MMP-1. The MMP-1 digestion was initiated by mixing the peptide with MMP-1 dissolved in double strength pH 7 buffer and incubated at 32 °C. The pH of the reaction mixture after mixing was 7, which is the optimal pH for the function of MMP-1. Both the noncleaved peptides and the digestion fragments were well resolved by SDS-PAGE (Figure 4B). As expected, the Colt3_0 peptide is largely resistant to MMP-1 digestion. Since the enzymatic reaction is promoted by the concentration of the substrate, the digestion of Colt3_0 was carried out at a higher peptide concentration than the other two peptides to estimate the higher limit of the resistance of the peptide to MMP-1. The two MMP-1 digestion fragments of Colt3_1 were clearly resolved with the N20 migrates to just below the 35 kDa marker but above 30 kDa (yellow arrow, Figure 4B), and the C7 below 15 kDa (orange arrow, Figure 4B). The noncleaved peptide of the Col3_1 peptide (Ctrl), which migrated in a similar fashion as the Ctrl of Colt3_0 and Colt3_2 (also see Figure 2A), was not included in the gel picture of Figure 4 because it was run on a lane separated from the group of digested samples; the band density of the Ctrl (or 0 min) lane was included for the densitometry analysis to get the kinetic data. There appears to be a very faint band in the low molecular weight region in all three peptides (red arrows Figure 4B), which remains constant over the course of MMP-1 incubation. This band likely represents impurity and was not included in the densitometry analysis shown in Figure 4C.

The multitude of the digestion fragments of Colt3_2 can be identified using the N20 and C7 fragments of Colt3_1 as references. The band below N20 can be unambiguously assigned to the C19 fragment (light-blue arrow, Figure 4B). The assignment of the doublet just above the 15 kDa marker, however, is ambiguous. The upper band of the doublet is likely the M12 (purple arrow, Figure 4B) generated by the cleavage at both MMP-1 sites, while the lower one is the N8 (dark-blue arrow, Figure 4B). It is curious why N8 migrated way above its molecular weight of 8 kDa to a location above 15 kDa especially compared to the C7 fragment, which is clearly below the 15 kDa marker. The higher proline content of N8 (28%) over that of C7 (20%) may contribute to the slowed migration on gel. N8 has the highest relative content of Pro among all fragments, which is also consistent with the observation that the lower band of the doublet is relatively poorly stained compared to the others since the neutral Pro residues do not bind the dye molecule in Coomassie blue. The alternative interpretation is that the N8 formed a dimer due to the Cys residues at the N-terminal Cys-knot sequence. In this case, there is a chance that the dimer N8 migrated to a location higher than the M12 fragment. If so, the dimer N8 should be assigned as the upper band of the doublet. While there is a possibility for the N8 to dimerize, it is hard to see why all of the N8 fragments are in the dimer form while in general the dimer only accounts for a small population of other fragments in the similarly treated samples (with the addition of the reducing reagent DTT). Additional study in connection with in-gel-digestion and mass-spec sequencing may resolve the ambiguities about the two bands. The definitive identification of the digested fragments, however, is not necessary of the kinetic analysis. The MMP-1 digestion of the peptides can be sufficiently characterized using only the time-dependent changes of the noncleaved peptides, as described in the next paragraph. Nonetheless, the identification of the digestion fragments of both Colt3_1 and Colt3_2 peptides, albeit not perfect, is a clear indication that the degradation is caused by MMP-1 cleavage at the expected site(s).

The rate of the MMP-1 digestion was analyzed by the relative change of the density of the bands of noncleaved peptides and their oligomers (Figure 4C). The initial rate of the enzyme digestion was determined by the linear fit of the data during the first 10 min of reaction where the curve appears to be linear. The initial rate of MMP-1 digestion of Colt3_1 is about 0.33 μM/min, while the initial rate of 0.49 μM/min for Colt3_2 at the same concentration is about 48% higher. The Colt3_0 peptide is significantly more resistant to MMP-1; the slope of the linear fit of Colt3_0 data during the first 60 min results in an initial rate of only 1.2 × 10^–3^ μM/min, about two orders magnitude lower than the other two peptides. All kinetic assays were carried out in duplicates with reproducible results.

Having only one MMP-1 digestion site, the kinetics of enzymatic digestion of Colt3_1 can be modeled by the Michaelis–Menten model. The *K*_M_ and *k*_cat_ of the reaction were estimated to be 13.23 μM and 5.86 min^–1^, respectively, based on the Lineweaver–Burk plot of the initial rates at four different substrate concentrations (Figure 4D). These estimations should be treated as the first approximation considering it is based on only four data points due to the limited availability of the peptides and from experiments with high inherent noise levels. It is, nonetheless, interesting to see that the *K*_M_ value is comparable with the 15 μM value estimated for another bacterial expressed collagen-mimetic peptide SC2#3.^48^ Peptide SC2#3 is a bacterial collagen-derived peptide having the 18-residue MMP-1 recognition site embedded at the center of the peptide. The value of *k*_cat_ for Colt3_1, however, is more than a magnitude lower compared to that of SC2#3. On the contrary, the *k*_cat_ for Colt3_1 is in the right ballpark with the values using tissue-derived type III collagen or a recombinant type III collagen expressed in as the substrate,^29,31,60^ although the *K*_M_ of Colt3_1 is about a magnitude higher than both in comparison. Both Colt3_1 and SC2#3 are about 2/3 the size of the native type III collagen; both are also lacking the Hyp since the bacterial expression system does not have the capability for the relevant post-translational modifications of eukaryotic cells. There is a Gly-Pro-Pro tripeptide immediately to the MMP-1 binding site at both the N- and C-terminals. The Pro residues in the Y-position of the two tripeptides are likely all hydroxylated to Hyp in type III collagen. The Pro in the N-terminal most triplet Gly-Ala-Pro of the MMP-1 binding site is also likely hydroxylated. While the overall low content of Pro and Hyp in the MMP-1 digestion site is frequently attributed to the local structural flexibility of the triple helix and rendering the particular Gly^846^–Ile^847^ bond to being susceptible to MMP-1 among several other Gly-Ile sites on collagen, it is not clear if the Hyp residues in the immediate vicinity facilitated the binding of the enzyme to the specific site and resulted in the lower *K*_M_ for the native collagens in comparison.

The *k*_cat_ of MMP-1 depends on a relatively unstable local structure of the substrate around the scissile bond.^61–64^ Colt3_1 has a high sequence homology to native type III collagen in the region surrounding the MMP-1 binding site. Its apparent *T*_m_ value of 44.3 °C is, however, a few degrees higher than the 36–37 °C of native collagens. This higher apparent *T*_m_ may translate to a reduced local flexibility of the MMP-1 digestion site and lead to a lower *k*_cat_. Thus, by increasing the overall stability of the Colt3 peptides, we may have inadvertently also increased their overall resistance to MMP-1. The bacterial collagen-derived SC2#3, on the other hand, has an apparent *T*_m_ similar to that of the native collagen, but a *k*_cat_ value about a magnitude higher.^48^ It remains interesting to find out if and how the variations in the sequence context of MMP-1 digestion site in SC2#3, especially the high content of charged residues,^65^ accelerated the activity of MMP-1. The discrepancies in the kinetic parameters can also be caused by the variations in the preparations of MMP-1 and the purity of the substrate. The MMP-1 enzyme is known for being unstable, and the activity can vary between batches. The experiments reported here are carried out using the same batch of activated MMP-1 purchased from Sigma.

### 4 The MMP-1 Susceptibility of the Colt3 Peptides in the Fibrillar Form

The ability to characterize the MMP-1 digestion of the Colt3 peptides in the fibrillar form provides a more direct understanding of the MMP-1 susceptibility of the materials during applications. For these experiments, the peptides had been incubated at 32 °C in fibrillogenesis buffer for 20–24 h to ensure the fibril formation before the addition of MMP-1. The MMP-1 digestion of the Colt3 peptides generated the same fragments when monitored by SDS-PAGE (Figure 5A), but at a much slower rate. The approximated initial rates of MMP-1 digestion of Colt3_1 (5.1 μM) and Colt3_2 (6.1 μM) are 0.026 and 0.040 μM min^–1^, respectively (Figure 5B); both are about 10× slower compared to the case when the substrates were in the triple-helix conformation as described above. The fibrillar form has clearly impeded the enzyme binding and/or digestion. The nearly identical 10× reduction in initial rates for both Colt3_1 and Colt3_2 peptides indicate the fibrillar structure impact the two digestion sites of Colt3_2 in a similar manner, if we attribute the differences in the degradation solely to the action of MMP-1; the self-assembly did not preferentially obscure the accessibility of one binding site over the other. A lower degree of digestion of the fibrillar Colt3_0 peptide was also observed. Interestingly, the estimated initial rate of 1.06 × 10^–3^ μM min^–1^ for the digestion of fibrillar Colt3_0 by MMP-1 is similar to the one shown in Figure 4C. No matter what factor(s) made the Colt3_0 susceptible to MMP-1, this property does not seem to be affected by the fibrillization of the peptide. Because of the longer incubation time for digestion of fibrillar structures, the digested fragments of Colt3_0 were accumulated enough to be seen on the gel and the migration rates of the fragments seemed to resemble those of Colt3_2 peptides (Figure 5A). It is possible that the Ile-to-Ala replacements at the scissile bond did not completely block the digestion of MMP-1 (Figure 1). Alternatively, this slow degradation may reflect a nonspecific digestion of the peptide by MMP-1, which is indifferent to the conformation of the substrates.

**Figure 5:**
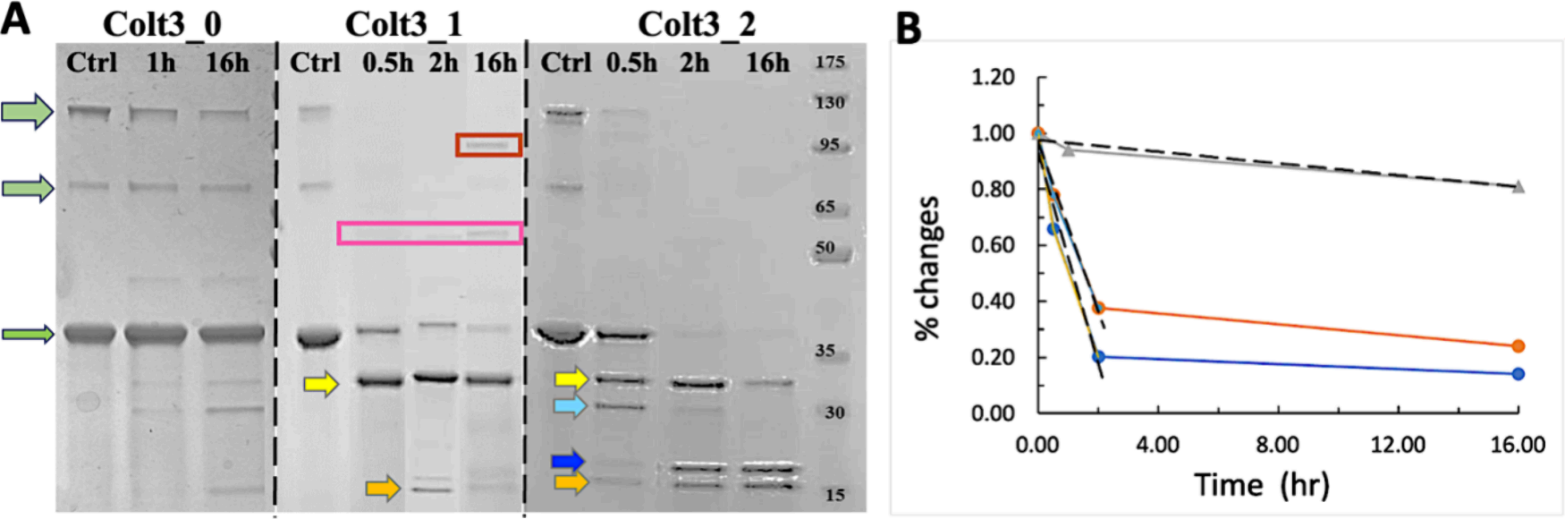
The MMP-1 digestion of the fibrillar form of Colt3 peptides. (A) Digestion fragments monitored by SDS-PAGE. The three green arrows in increasing width represent, respectively, the monomer, dimer, and trimer forms of noncleaved peptides (Ctrl). The digestion fragments are identified by the arrows in the same color scheme as in Figure 4A. The red and pink boxes identify the oligomer forms of the digested peptides (see text). The last lane is molecular marker in kDa. The concentration of MMP-1 is 200 nM. The vertical dashed line marks the boundary of gels. (B) Time course of MMP-1 degradation of fibrillar form Colt3_0 (gray), Colt3_1 (orange), and Colt3_2 (blue). The initial rates of the reaction were estimated by the linear fit shown as dashed line. The solid lines connecting the data points are included as a guide to the eye. The concentrations of Colt3_1 and Colt3_2 are 5.1 and 6.1 μM, respectively, and that of Colt3_0 is 14.7 μM.

It is difficult to carry out a quantitative analysis of the proteolysis of collagen in the fibrillar form by MMP-1, or by any enzyme for that matter. The digestion of fibrillar Colt3 peptides is carried out in the solution phase, where the fibrillogenesis is governed by an equilibrium between the triple helices and the self-associated fibrils. Because of the inherent high degree of heterogeneity in both the fibril diameter and fibril length, we were not able to get a reliable estimation of the association constant of the fibrillogenesis of the peptides. We do expect, however, that the degree of fibril formation is similar among the three peptides due to their sequence homology and similar fibril conformations (Figure 3). The differences of the three peptides are limited to two to four residues in a peptide consisting of over 200 residues in the triple-helix domain. The impact of these substitutions on the fibrillogenesis, which is a process driven mostly by the interactions of charged residues and hydrophilic residues,^66,67^ is expected to be minimal. The inhibited MMP-1 rate likely reflected both the impact of the reduced concentration of the substrate in solution due to the fibril assembly, and the intrinsic higher resistance of the fibrillar structures to the enzyme; the latter may further depend on the thickness and the length of the fibrils. The sample heterogeneity also prevented a meaningful estimation of the impact of either the diameter or the length of the fibrils on their interactions with MMP-1 in the solution phase. The tendency of gelation of collagen triple helices at higher concentrations further complicates the experimental approach to control the fibril diameter by varying concentrations and/or the incubation time in fibril-forming buffer and temperature.

The catalytic activity of MMP-1 is primarily directed by the specific amino acid sequences surrounding the scissile bond. The fibrillar structure, however, will affect the rate of digestion. An earlier study using tissue-derived collagen by Welgus and colleagues reported that the digestion rate of MMP-1 with the reconstituted fibrils as the substrate were slowed by 25–100 times compared to the cases using the triple helical collagen monomers as the substrate.^31^ In their study, the fibrillogenesis produced gels and the rate of digestion was estimated by the released digested fragments from the gels. Their study also faced the same uncertainties of the presence and/or the release of collagen triple helices in the gel phase. A more recent study using single-molecule technology reported a relatively high digestion rate of MMP-1 on collagen fibrils isolated from rate tail tendon, which is constituted mostly by type I collagen.^32,33^ The digestion rate was estimated by the events as MMP-1, which had already lashed onto the fibrils, moved along the tendon in a halted, unidirectional manner. Their digestion rate of 18 collagen/min cannot be directly compared with the rate observed by bulk study of fibrils in solution or in the gel phase since it does not include the binding reaction of MMP-1 on the substrate.

This initial work focuses on characterizing the susceptibility of the peptides to MMP-1 at a qualitative level; further study is underway to provide more precise quantitative analysis of the mechanisms of the digestion. The fibril-forming ability of the peptides also offers more leverage to optimize the functionality of the designed biomaterials. The low yield of the bacterial expressed peptides and the lack of Hyp have limited the scope of the current work, but these limitations are far from being unsurmountable. The yield can be increased significantly using a fermenter; several approaches are being tested to include the hydroxylation in a bacterial construct,^68,69^ or by expressing the designed peptides in a mammalian cell line. Another development is to utilize the heterotrimeric nucleation sequences to create heterotrimeric triple helices such as type I collagen.^70^ These new developments pave the way for the MRCs to become a robust and versatile biomaterial for bioengineering and biomedical applications.

## Conclusions

In this work, we showed the feasibility to customize the functionality of collagen-mimetic biomaterials using designed, fibril-forming peptides. Having nearly identical amino acid sequences, the three Colt3 peptides are designed to have similar biological functions and properties except the susceptibility to MMP-1. By including an additional MMP-1 recognition site, the turnover rate of the Colt3_2 peptide is increased by nearly 48% in both the triple helical form and the fibrillar form compared to that of peptide Colt3_1, which carries only one canonical MMP-1 digestion site. The fast and also tunable susceptibility of the collagen-mimetic materials offers a way to minimize the fibrosis and the development of scar tissues when used for wound healing. The Colt3_0 peptide, on the other hand, is made significantly more resistant to MMP-1—a desirable feature for collagen-based medical devices where the fast, innate turnover rate of collagenous materials limits their effectiveness. Combing the protein design with biochemical and enzymatic studies, we demonstrated the potential of MRCs as biomaterials for regenerative medicine with tailor-made functionality.

## Supplementary Material



## Notes

Complete amino acid sequences of peptides: Colt3_0, Colt3_1, and Colt3_2 (PDF)

## Notes

Dr. Jui Shivaji Chaugule: ProViZigen, 160 Convent Ave., at 140th st., Steinman Hall T313, New York, NY 10031

## Abbreviations

CD: circular dichroism
DTT: dithiothreitol
IPTG: isopropyl-β-d-thiogalactopyranoside
HPLC: high-performance liquid chromatography
Hyl: hydroxylysine
Hyp or O: hydroxyproline
LC-MS: liquid chromatography mass spectrometry
SDS-PAGE or SDS-gel: sodium dodecyl sulfate-polyacrylamide gel electrophoresis
TEM: transmission electron microscope
