# Supplementary material for: Designed Fibril-Forming Mini-Collagens Engineered to Exhibit up to Two Orders of Magnitude Differences in Rates of Matrix Metalloproteinase I Susceptibility
Source: Biomacromolecules. 2025 Jun 23;26(7):4040–50. doi: 10.1021/acs.biomac.5c00026 (PMC12264941; doi:10.1021/acs.biomac.5c00026)
Supplement: Supplementary file 1 [file bm5c00026_si_001.pdf]

# Designed Fibril-forming mini-collagens engineered to exhibit up to two orders of magnitude differences in rates of matrix metalloproteinase I susceptibility

*Jui Shivaji Chaugule*[1,2,†], *and Yujia Xu*[1,2] *

[1] The Graduate Center, Program of Biochemistry, The City University of New York, 365 5th Ave., New York NY 10016, United States

[2] Department of Chemistry, Hunter College of the City University of New York, 695 Park Ave., New York, NY 10065, United States

* Email: yujia.xu@hunter.cuny.edu

## Amino Acid Sequences of Peptides

Colt3_0

GSGPCCGPPGPPGPPGPPGAR GEP GAR GLP GPP GSE GAP GPR GPS GEP GKM GPR GPK GET GAP
GEP GVR GPK GDA GQP GEK GEP GEQ GPR GAP GPL GAA GPT GAR GLA GPP GAP GPR GEP GPQ
GVRGTPGPPGPPGPPGPPGAR GEP GAR GLP GPP GSE GAP GPR GPS GEP GKM GPR GPK GET GAP
GEP GVR GPK GDA GQP GEK GEP GEQ GPR GAP GPL GAA GPT GAR GLA GPP GAP GPR GEP GPQ
GVRGPPGPPGPPGPP GPCCGSGYIPEAPRDGQAYVRKDGEWVLLSTFL

Colt3_1

GSGPCCGPPGPPGPPGPPGAR GEP GAR GLP GPP GSE GAP GPR GPS GEP GKM GPR GPK GET GAP
GEP GVR GPK GDA GQP GEK GEP GEQ GPR GAP GPL GAA GPT GAR GLA GPP GAP GPR GEP GPQ
GVRGTPGPPGPPGPPGPPGAR GEP GAR GLP GPP GSE GAP GPR GPS GEP GKM GPR GPK GET GAP
GEP GVR GPK GDA GQP GEK GEP GEQ GPR GAP GPL GAI GIT GAR GLA GPP GAP GPR GEP GPQ
GVRGPPGPPGPPGPPGPCCGSGYIPEAPRDGQAYVRKDGEWVLLSTFL

Colt3_2

GSGPCCGPPGPPGPPGPPGAR GEP GAR GLP GPP GSE GAP GPR GPS GEP GKM GPR GPK GET GAP
GEP GVR GPK GDA GQP GEK GEP GEQ GPR GAP GPL GAI GIT GAR GLA GPP GAP GPR GEP GPQ
GVRGTPGPPGPPGPPGPPGAR GEP GAR GLP GPP GSE GAP GPR GPS GEP GKM GPR GPK GET GAP
GEP GVR GPK GDA GQP GEK GEP GEQ GPR GAP GPL GAI GIT GAR GLA GPP GAP GPR GEP GPQ
GVRGPPGPPGPPGPPGPCCGSGYIPEAPRDGQAYVRKDGEWVLLSTFL
